# Tocilizumab in improving the hemodynamics of septic children with vasoplegia shock

**DOI:** 10.1186/s13054-025-05512-6

**Published:** 2025-06-20

**Authors:** En‑Pei Lee, Jainn-Jim Lin, Shih‑Hsiang Chen, Han‑Ping Wu

**Affiliations:** 1 Division of Pediatric Critical Care Medicine, Department of Pediatrics, Chang Gung Memorial Hospital at Linko, Kweishan, Taoyuan, Taiwan; 2 College of Medicine, Chang Gung University, Taoyuan, Taiwan; 3 Divisions of Hematology and Oncology, Department of Pediatrics, Chang Gung Memorial Hospital, Chang Gung University, Taoyuan, Taiwan; 4 Department of Pediatrics, Chiayi Chang-Gung Memorial Hospital, No. 6, W. Sec., Jiapu Rd, Puzi City, Chiayi County 613 Taiwan

Sepsis remains a leading cause of pediatric morbidity and mortality worldwide. Septic shock, a progression of sepsis characterized by profound cardiovascular dysfunction, carries mortality rates as high as 40–80% and often results in long-term neurocognitive deficits. In patients with septic shock, vasoplegia is the worse presentation of hemodynamics which related higher mortality. Vasoplegia is an abnormally low systemic vascular resistance (SVR) that is manifest as profound hypotension or the requirement for therapies to avoid this, in the presence of a normal or increased cardiac output [1].

IL-6 plays a pivotal role in sepsis-induced endothelial injury, promoting vascular permeability, myocardial dysfunction, and ultimately vasoplegia. Tocilizumab, an IL-6 receptor blocker, has shown promise in modulating inflammatory cascades in various hyperinflammatory states. This study investigates whether tocilizumab improves hemodynamics and outcomes in pediatric patients with septic shock and vasoplegia.

We retrospectively analyzed pediatric patients with septic shock admitted to the PICU of Chang Gung Children’s Hospital from January 2018 to February 2025. Inclusion criteria were a diagnosis of septic shock requiring vasoactive support.

Pediatric septic shock was defined as cardiovascular dysfunction (hypotension, need for vasoactive agents) or impaired organ perfusion and therapeutic strategies were based on the 2017 American College of Critical Care Medicine [2]. From 2022 onward, patients with elevated IL-6 levels received a single dose of tocilizumab early during shock. Patients from 2018 to 2021 served as historical controls without tocilizumab therapy.

Hemodynamic monitoring was performed using a pulse index continuous cardiac output (PiCCO) system (Pulsion Medical Systems, Munich, Germany) or electrical cardiometry (ICON, Osypka Medical GmbH, Berlin, Germany), depending on the study period. We collected serial cardia index (CI), systemic vascular resistance index (SVRI) and vasoactive-inotropic score (VIS) during the first 72 h of PICU admission. Serum IL-6 levels were measured serially at days 0, 3, 5, and 7 using the Elecsys immunoassay. Vasoplegia was defined as the SVRI lower than normal range without the support of vasopressor [3]. To quantify the severity of vasoplegia and monitor its clinical progression, we developed the Vascular Reactivity Index (VRI), defined as SVRI/VIS (Systemic Vascular Resistance Index divided by Vasoactive-Inotropic Score). A lower VRI reflects greater vasoplegia severity and signals the need for more intensive hemodynamic support. In our cohort, VRI demonstrated favorable prognostic accuracy, with an average area under the receiver operating characteristic curve (AUC) exceeding 0.80 for predicting mortality in pediatric septic shock with vasoplegia [4].

The baseline characteristics had no significantly difference between the tocilizumab (*n* = 31) and control (*n* = 33) groups. Bloodstream infection was the predominant etiology in both groups. Tocilizumab-treated patients demonstrated significantly improved outcomes: 28-day mortality was reduced from 54.5 to 19.3% (*p* < 0.05), and shock duration was shorter. Hemodynamically, these patients exhibited faster normalization of SVRI and reduction in VIS. As shown in Table 1, patients treated with tocilizumab exhibited a significantly faster reduction in VIS, indicating earlier weaning from vasopressors, particularly norepinephrine and epinephrine. VRI, a surrogate marker for vascular recovery, improved more rapidly in the treatment group. IL-6 levels declined in parallel with VRI and SVRI improvements following tocilizumab administration. Kaplan-Meier survival analysis confirmed a significantly higher survival rate in the tocilizumab group (log-rank *p* < 0.01).

This study suggests that early IL-6 inhibition with tocilizumab may reverse vasoplegia and reduce mortality in pediatric septic shock. By attenuating IL-6-driven endothelial dysfunction and capillary leakage, tocilizumab likely contributes to vascular tone restoration. Notably, the intervention was effective even in immunocompromised patients, consistent with prior observations in febrile neutropenic children [5]. Previous anti-cytokine therapies targeting IL-1, TNF-α, and IL-10 have largely failed to improve septic shock outcomes. The pathophysiological role of IL-6 in sepsis includes promoting the overproduction of vascular endothelial growth factor (VEGF), which enhances angiogenesis and markedly increases vascular permeability. The resulting capillary leak contributes to interstitial edema, elevating tissue pressure and impairing organ perfusion. Moreover, IL-6 directly impairs myocardial function by weakening papillary muscle contraction, thereby contributing to septic cardiomyopathy. Consistently, elevated IL-6 levels correlate with worse clinical outcomes, including progression to septic shock and increased mortality. Tocilizumab, a monoclonal antibody targeting both membrane-bound and soluble IL-6 receptors, inhibits these downstream pathways, thereby preserving endothelial integrity and potentially mitigating multi-organ damage. Importantly, no significant increase in secondary infections was observed in this cohort, although larger trials are needed to confirm safety. The study is limited by its retrospective design and single-center setting. Nevertheless, the consistent hemodynamic benefit and survival advantage observed support further investigation in randomized trials.

In pediatric patients with vasoplegia septic shock, adjunctive tocilizumab is associated with improved hemodynamics, shortened shock duration, and reduced mortality. IL-6 may serve as both a biomarker and therapeutic target in this population. Prospective multicenter trials are urgently needed to validate these findings.

**Table 1:** Demographics and initial parameters between septic patients with vasoplegia shock treated with and without Tocilizumab

Variables	Patients Treated Without Tocilizumab (*n* = 33)	Patients Treated with Tocilizumab (*n* = 31)	*p*-Value
Age (years)	12.2 ± 4.3	11.1 ± 6.7	0.454
Sex (male), n (%)	16 (48.4)	15 (48.3)	
Weight (kg)	35.6 ± 14.8	39.7 ± 25.7	0.443
Underlying, n (%)	22 (66.6)	24 (77.4)	
PRISM III score	19.1 ± 4	17.8 ± 4.2	0.217
Laboratory examination, median (IQR)			
WBC (u/L)	10,300 (9,600 − 17,100)	3,600 (100 − 12,300)	0.341
Platelet (∗10^3^ )	172 (81–455)	95 (7–204)	0.152
Creatinine (mg/dL)	0.9 (0.46–1.84)	0.67 (0.35–1.1)	0.441
Peak procalcitonin (ng/mL)	22.7 (4.9–71)	26.2 (6.4–80)	0.42
Hemodynamics (mean ± SD)			
Day 1 CI (L/min/m^2^)	4 ± 1.2	4.2 ± 0.9	0.568
Day 2 CI (L/min/m^2^)	4.2 ± 1	3.9 ± 0.4	0.363
Day 3 CI (L/min/m^2^)	3.6 ± 0.9	3.2 ± 0.4	0.163
Day 1 SVRI (dyn×sec/cm^5^/m^2^)	1100 ± 510	954 ± 250	0.06
Day 2 SVRI (dyn×sec/cm^5^/m^2^)	1295 ± 538	1647 ± 189	0.009*
Day 3 SVRI (dyn×sec/cm^5^/m^2^)	1714 ± 657	2105 ± 286	0.039*
Day 1 VIS	44.6 ± 26.8	39.5 ± 24.7	0.58
Day 2 VIS	36 ± 28	22 ± 15	0.047*
Day 3 VIS	28.4 ± 22.4	4.8 ± 4.1	0.001*
Day 1 VRI	42.3 ± 6.1	39.2 ± 8.3	0.757
Day 2 VRI	82.1 ± 22.4	149.2 ± 30.1	0.08
Day 3 VRI	156 ± 56.1	952.7 ± 177.7	0.001*
Outcomes			
Shock duration (hours)	240 (120–336)	96 (48–120)	< 0.001*
Admission stay (days)	20 (15–30)	13 (12–16)	0.002*
Mortality, n (%)	18 (54.5)	6 (19.3)	0.004*

PRISM, Pediatric Risk of Mortality; WBC, white blood cell; CI, cardiac index; SVRI, systemic vascular resistance index; VIS, vasoactive-inotropic score; VRI, vascular reactivity index; ICU, intensive care unit. * Statistical significance was set at *p* < 0.05

## Data Availability

The datasets used and analyzed during the current study are available from the corresponding author on reasonable request.
